# Screen-Printed Carbon Electrodes with Cationic Cyclodextrin Carbon Nanotubes and Ferrocenyl-Carnosine for Electrochemical Sensing of Hg(II)

**DOI:** 10.1021/acsanm.3c03480

**Published:** 2023-09-11

**Authors:** Chiara Abate, Giulia Neri, Angela Scala, Placido Giuseppe Mineo, Enza Fazio, Antonino Mazzaglia, Alex Fragoso, Ottavia Giuffrè, Claudia Foti, Anna Piperno

**Affiliations:** † Department of Chemical, Biological, Pharmaceutical and Environmental Sciences, University of Messina, Viale F. Stagno d’Alcontres 31, Messina 98166, Italy; ‡ Department of Chemical Sciences, University of Catania, Viale A. Doria 6, Catania 95125, Italy; § Department of Mathematical and Computational Sciences, Physical Sciences and Earth Sciences, University of Messina, Viale F. Stagno d’Alcontres 31, Messina 98166, Italy; ∥ National Council of Research, Institute for the Study of Nanostructured Materials (CNR-ISMN), URT of Messina c/o Department of Chemical, Biological, Pharmaceutical and Environmental Sciences, University of Messina, Viale F. Stagno d’Alcontres 31, Messina 98166, Italy; ⊥ Inferfibio Research Group, Departament d’Enginyeria Qúmica, Universitat Rovira i Virgili, Avinguda Päsos Catalans 26, Tarragona 43007, Spain

**Keywords:** cationic cyclodextrin carbon nanotubes, ferrocenylcarnosine, modified screen-printed carbon electrodes, voltammetry, mercury sensor

## Abstract

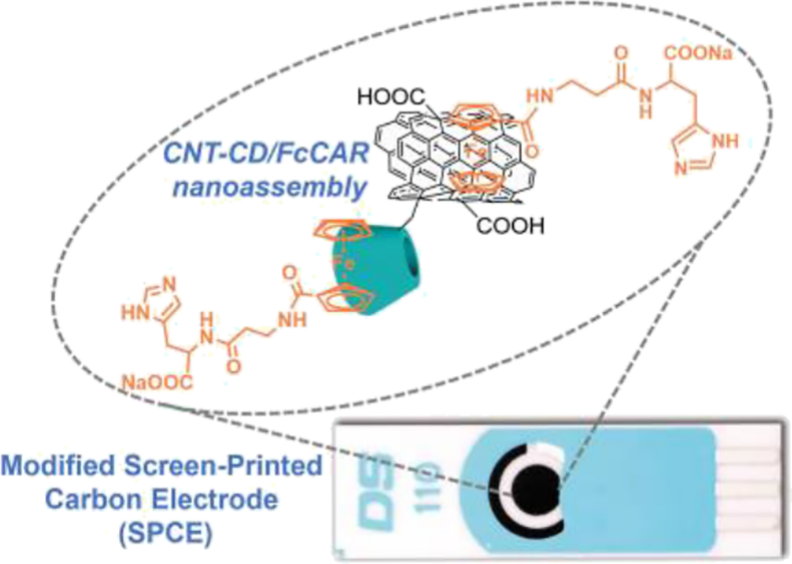

The study reports the use of nanoassembly based on cationic cyclodextrin carbon nanotubes (CNT-CDs) and ferrocenylcarnosine (FcCAR) for electrochemical sensing of Hg(II) in aqueous solution. β-cyclodextrins (CDs) were grafted onto CNTs by a click chemistry reaction between heptakis-(6-azido-6-deoxy)-β-cyclodextrin and alkyne-terminated CNTs. The cationic amine groups on the CD units were produced by the subsequent reduction of the residual nitrogen groups. The chemical composition and morphology of CNT-CDs were analyzed by X-ray photoelectron spectroscopy, scanning electron microscopy, and thermogravimetric analysis. A *N*,*N*-dimethylformamide dispersion of CNT-CDs was cast on the surface of screen-printed carbon electrodes (SPCEs), and the electrochemical response was evaluated by cyclic voltammetry (CV) using [Fe(CN)_6_]^3–^ as the redox probe. The ability of SPCE/CNT-CD to significantly enhance the electroactive properties of the redox probe was combined with a suitable recognition element (FcCAR) for Hg(II). The electrochemical response of the CNT-CD/FcCAR nanoassembly was evaluated by CV and electrochemical impedance spectroscopy. The analytical performance of the Hg(II) sensor was evaluated by differential pulsed voltammetry and chronoamperometry. The oxidative peak current showed a linear concentration dependence in the range of 1–100 nM, with a sensitivity of 0.12 μA/nM, a limit of detection of 0.50 nM, and a limit of quantification of 1 nM.

## 1 Introduction

Electrochemical sensors are considered useful analytical tools because of their sensitive and selective detection of analytes, rapid response, ease of operation, and low cost of reagents and instrumentation. Moreover, in the past decade, nanotechnology-based approaches have advanced significantly, leading to enhanced analytical response and miniaturization of devices.^1−3^ In particular, modification of the electrode surface with carbon-based nanomaterials is a widely exploited strategy for the immobilization of recognition elements. The additional layer of nanomaterial that alters the shape and structure of the electrode surface can increase the electrical conductivity and surface area, improving sensitivity. Several carbon-based nanomaterials have been proposed in the literature for electrode surface modification, including carbon nanotubes (CNTs), graphene, carbon onions, graphene quantum dots, etc., both in their native form and as chemically functionalized derivatives.^4−9^

Multiwalled CNTs are considered rolled sheets of graphene that form single- or multiwalled seamless cylinders with diameters ranging from a few to hundreds of nanometers (SW- and MW-CNTs, respectively). They typically have a length-to-diameter ratio greater than 10^6^ and exhibit exceptional electrical, thermal, mechanical, and optical properties.^10,11^ In our ongoing research program aimed at the development of high-performance multifunctional materials, we studied the biocompatibility, processability, and colloidal stability of carbon-based nanomaterials covalently functionalized with cationic β-cyclodextrins (CDs).^12^ The latter, grafted onto the carbon nanomaterials, improved the recognition properties by providing lipophilic interaction sites (i.e., CD cavities) and electrostatic interaction sites due to the outer rims.

**Figure 1:**
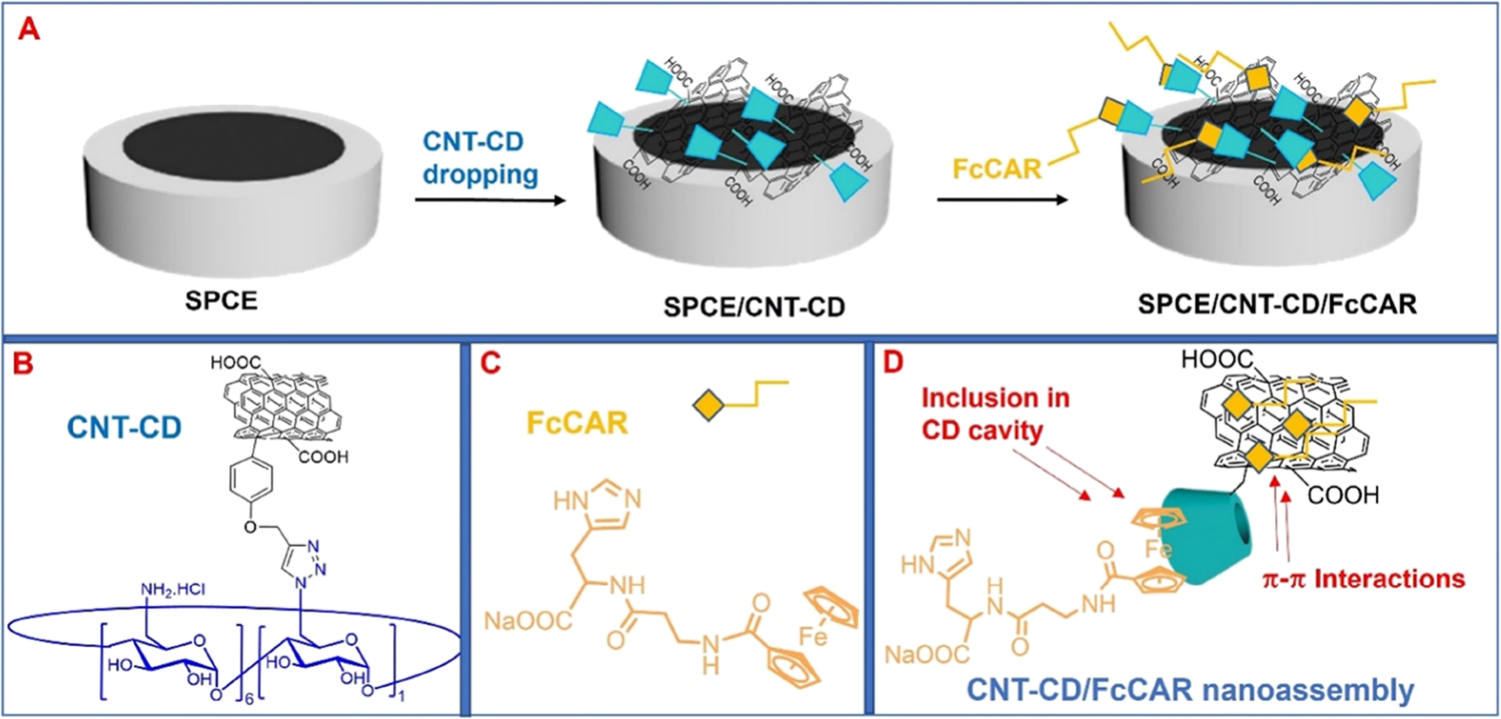
(A) Representative fabrication route of electrochemical sensors based on SPCEs modified with CNT-CD and FcCAR. (B, C) Chemical structures of cationic CNT-CD and FcCAR, respectively. (D) CNT-CD/FcCAR nanoassembly and molecular recognition sites between CNT-CD and FcCAR.

In line with this research topic, here, we describe the synthesis and characterization of cationic β-cyclodextrin-functionalized CNTs (CNT-CDs).^13−17^ The ability of CDs to form stable host-guest inclusion complexes^17,18^ was combined with the design of a suitable recognition element (ferrocenylcarnosine (FcCAR)^19,20^) that can act as a guest, allowing the fine-tuning of specific features for electrochemical sensing. FcCAR was designed considering three main characteristics: (1) the electroanalytical properties of the Fc unit; (2) the high affinity of CAR for heavy metals^19,20^; and (3) the high affinity of CD cavities for the Fc unit (*K* ≈ 10^3^ M),^21^ which allows efficient inclusion by supramolecular interactions. Host–guest interactions between CNT-CD and FcCAR have been exploited to achieve nanoassembly for the modification of screen-printed carbon electrodes (SPCEs). The latter are innovative strips produced and very useful for electrochemical analysis in many fields (environmental, clinical, food) because of their low cost, ability to work with microvolumes, availability, and large-scale production. Figure 1 shows the chemical structures of CNT-CD and FcCAR, their molecular recognition sites, and the representative fabrication routes of SPCE/CNT-CD/FcCAR-based electrochemical sensors.

Considering the high affinity of FcCAR toward Hg(II),^19^ we propose the use of SPCE/CNT-CD/FcCAR as a mercury sensor. The new system showed very good analytical performance in Hg(II) detection, with a linear concentration range of 1–100 nM, sensitivity of 0.12 μA/nM, a limit of detection (LOD) of 0.50 nM, and a limit of quantification of 1 nM.

## 2 Materials and Methods

### 2.1 Synthesis of CNTs Modified with Cationic Amine CDs (CNT-CD)

CNT-Alk (**1**) was prepared as described in SI and according to the procedure we previously reported.^10^ 200 mg of CNT-Alk (**1**) was dispersed in 30 mL of DMF by sonication treatment, followed by the addition of heptakis-(6-azido-6-deoxy)-β-cyclodextrin (CDN_3_, **2**) (164 mg, 0.15 mmol), CuSO_4_ (30 mg, 0.17 mmol), and Na ascorbate (70 mg, 0.34 mmol). The reaction was left for 48 h at 80 °C under argon flow. The cooled mixture was diluted with water (200 mL) and filtered under vacuum (0.1 μm Millipore membrane). The residue was washed with water, ethanol, and methanol, collected, and dried at 60 °C to obtain 210 mg of CNT-CDN_3_ (**3**). Triphenylphosphine (463 mg, 1.76 mmol) was added to a dispersion of CNT-CDN_3_ (**3**) in DMF (195 mg, 9 mL), and the mixture was stirred at room temperature for 1 h. Afterward, the temperature was raised to 45 °C, 28% ammonia solution was added dropwise, and the reaction was allowed to stir at 45 °C for 24 h. The mixture was diluted with a water/EtOAc solution (1:1 v/v) and filtered under vacuum (0.1 μm Millipore membrane). The solid residue was washed with a chloroform/water/methanol solution (1:0.5:0.5) and finally with methanol. Then, CNT-CDNH_2_ (**4**) was dispersed in 4 mL of DMF and treated with an excess of 15% HCl solution to reach a pH value between 1 and 2. The mixture was stirred for 1 h at room temperature, vacuum filtered (0.1 μm Millipore membrane), washed with water and methanol, and dried at 60 °C to recover 150 mg of CNT-CD (**5**). Primary amine loading was measured spectroscopically using Kaiser’s colorimetric assay,^22^ the protocol for which is given in Supporting Information (S1). The amount of amine groups on CNT-CDs was estimated to be 0.607 mmol g^–1^, roughly consistent with 0.101 mmol g^–1^ of grafted CDs.

### 2.2 Physicochemical Characterization

X-ray photoelectron spectroscopy (XPS) was performed under ultrahigh vacuum (UHV) conditions using a UK Thermo Scientific K-Alpha spectrometer equipped with a monochromatic Al Kα source (*h*ν = 1486.6 eV) and a hemispherical analyzer. The constant-pass energy was set at 200 eV for the survey scans and 50 eV for the high-resolution XPS spectra.

Micro-Raman measurements were made with a Horiba XploRA spectrometer (Horiba Italia s.r.l.). Samples were excited using the 532 nm line of a solid-state laser, integrated for 50 s, and collected with a charge-coupled detector (CCD) using a microscope objective with 50× focal length.

The morphology of the samples was examined by coating a holey-copper grid with their suspensions and drying them in air at room temperature. SEM micrographs were recorded at an accelerating voltage of 30 kV using a Zeiss Gemini 2 scanning electron microscopy (SEM). UV–vis spectra were recorded by an Agilent model 8452 diode-array spectrophotometer in quartz cells (1 cm optical path) at room temperature using water as solvent. Thermogravimetric analyses were performed with Pyris TGA7 (PerkinElmer, Waltham, MA, USA) in the temperature range of 50–800 °C, under a nitrogen flow rate of 60 mL min^–1^ and a heating rate of 10 °C min^–1^.

### 2.3 Electrochemical Equipment and Measurements

All voltammetric measurements were performed in aqueous solutions of KCl (0.1 M) and at room temperature using a PC-controlled electrochemical workstation (PNT-10-Autolab) equipped with Metrohm DropSens (DRP-DSC70575) SPCEs (DRP-110). The SPCEs consist of a carbon working electrode (diameter of 4 mm) surrounded by carbon auxiliary and Ag/AgCl reference electrodes. Voltammograms were processed with General Purpose Electrochemical System (GPES) software, version 4.9 (Eco Chemie B.V.).

SPCEs were modified by drop-casting a homogeneous dispersion of CNT-CD in DMF (3 mg mL^–1^) prepared under sonication for 30 min (VEVOR sonic bath). To obtain a thin layer of CNT-CD, 1 μL of the dispersion was cast four times on the surface of the electrodes and dried in an oven at 80 °C in a DMF atmosphere for 30 min. This procedure was used to avoid the formation of the so-called coffee-ring effect, which gives rise to inhomogeneous films.^23^

The intrinsic electrochemical properties and voltammetric responses of SPCE/CNT-CD were evaluated by Cyclic Voltammetry (CV) using [Fe(CN)_6_]^3–^ (1 mM) in KCl solution (0.1 M). CV was performed in the potential window from −0.3 to 0.8 V vs. Ag/AgCl and at a scan rate of 0.1 V s^–1^. Electrochemical impedance spectroscopy (EIS) measurements were recorded in the frequency range of 100 kHz–0.1 Hz at a bias potential of +0.16 V and an AC amplitude of 5 mV using an equimolar mixture of 1 mM [Fe(CN)_6_]^4–^ and [Fe(CN)_6_]^3–^ in KCl (0.1 M).

The electroactivity of FcCAR (1 mM) in aqueous solutions of KCl (0.1 M) was evaluated on SPCE/CNT-CD by CV in the potential range of 0.0 to 0.5 V vs. Ag/AgCl. Moreover, the analytical performance of SPCE/CNT-CD/FcCAR for the detection of Hg(II) was further investigated by DPV (in the same potential range of 0.0 to 0.5 V vs. Ag/AgCl, potential step: 10 mV, and amplitude: 100 mV) and by chronoamperometry at +0.38 V vs. Ag/AgCl while keeping the solution under gentle stirring (150 rpm).

## 3 Results and Discussion

Recently, we investigated the electrochemical behavior of FcCAR with divalent metal cations on bare SPCEs to exploit the sensing ability of FcCAR in the development of electrochemical sensors.^19^ These studies revealed the ability of FcCAR to act as a part of chemical recognition of the electrochemical probe by interacting with target ions, particularly Hg(II), and converting the chemical interactions to a measurable signal. To improve the current response and sensitivity of the SPCE, the working electrode was modified with a dispersion of CNT-CD.

### 3.1 Synthesis and Characterization of CNTs Modified with Cationic Amine CDs (CNT-CD)

**Figure 2:**
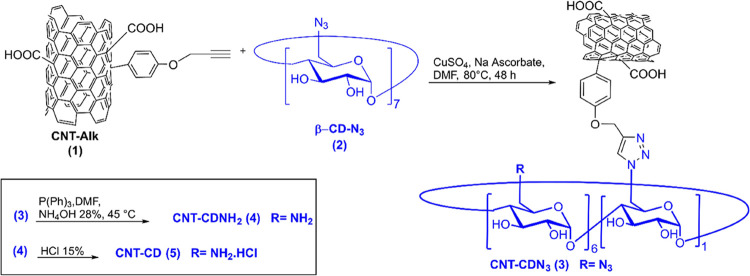
Schematic representation of the synthesis of CNT-CDs.

**Figure 3:**
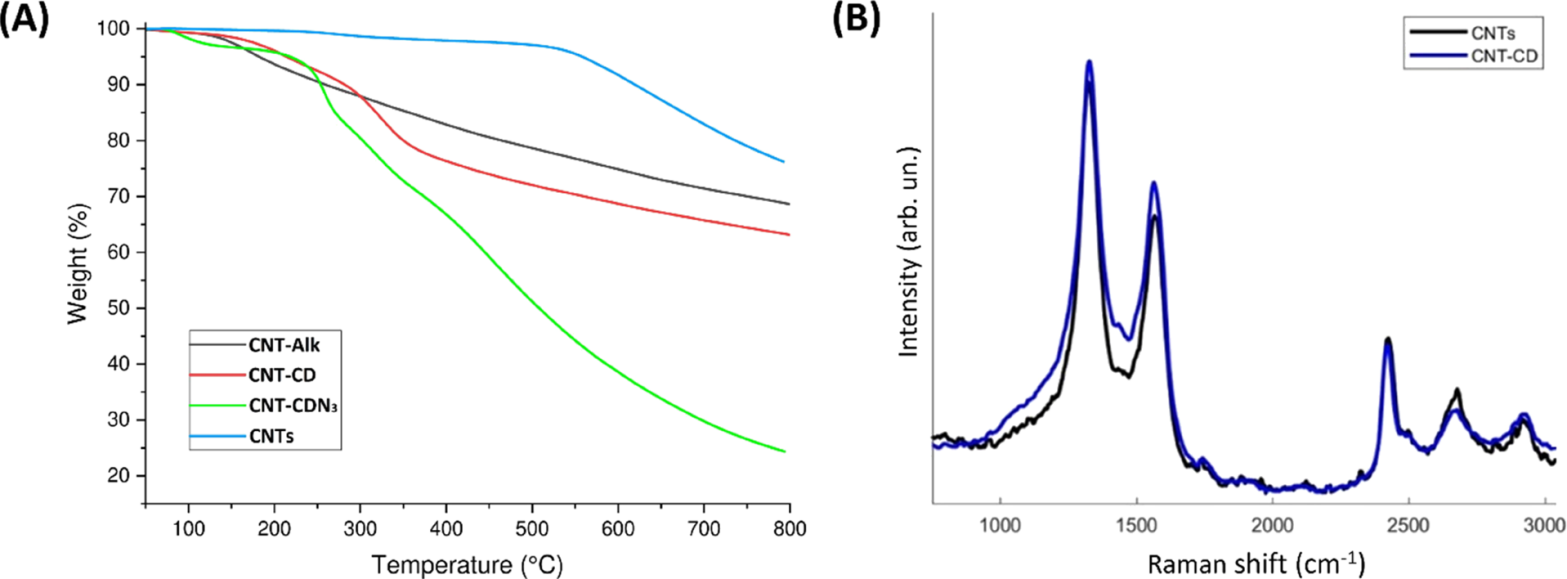
(A) TGA profiles of CNTs (blue line), CNT-Alk (black line), CNT-CDN_3_ (green line), and CNT-CD (red line) in nitrogen atmosphere. (B) Raman spectra of CNT-CDs compared with pristine CNTs.

**Figure 4:**
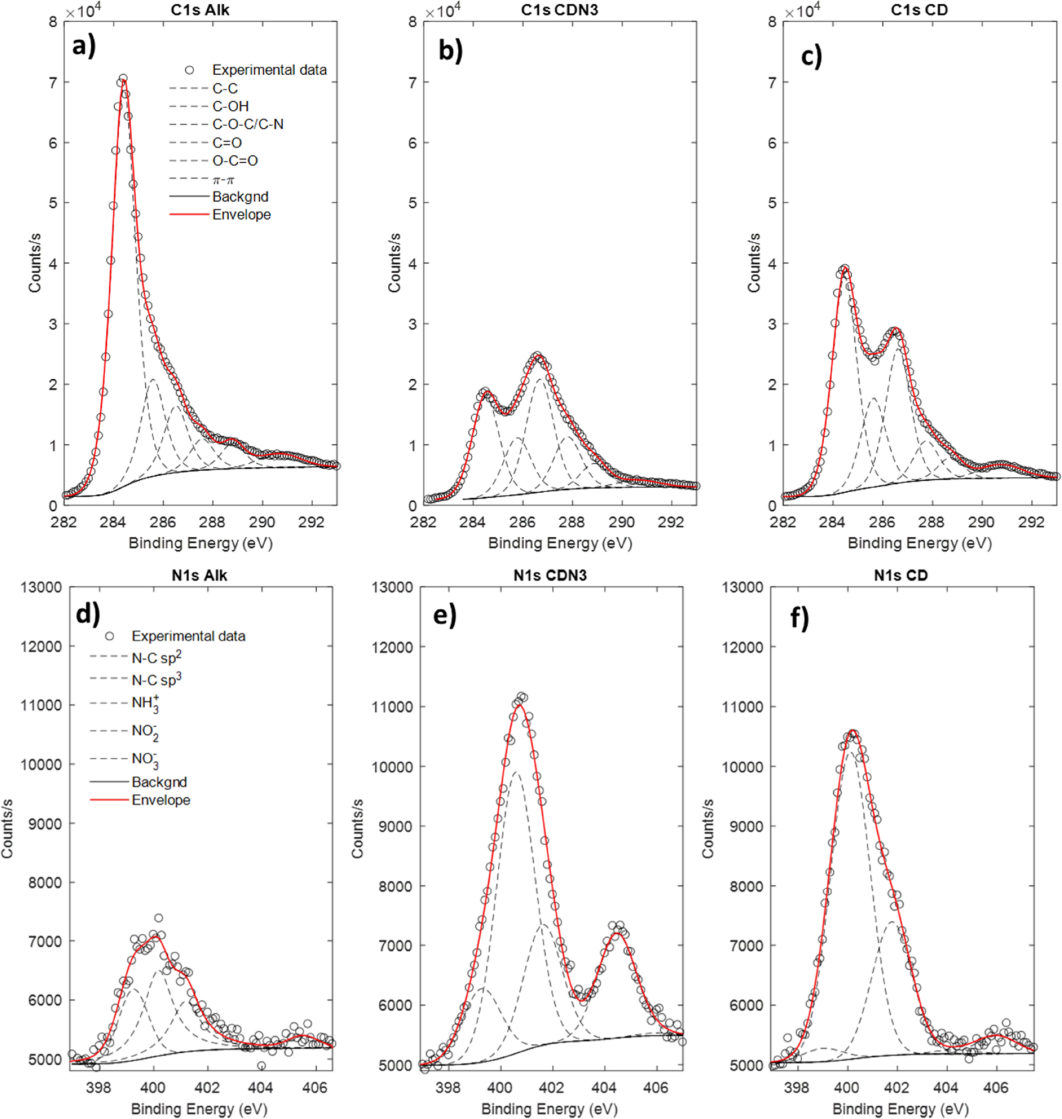
High-resolution deconvoluted XPS profiles of C 1s (a–c) and N 1s (d–f).

To produce hydrophilic CNTs endowed with specific recognition ability toward the ferrocenyl moiety of FcCAR, double functionalization of the surfaces of commercially available multiwalled CNTs was designed (Figure 2). The hydrophilic characteristics of commercial CNTs were improved by oxidation under acidic conditions (HNO_3_/H_2_SO_4_).^10^ Acid treatment produced CNTs with short open ends that have carboxyl groups on both the wall surface and the open ends.^11^ The second functionalization step provides the terminal alkyne-reactive moieties on CNTs (CNT-Alk, **1**) used for the binding of heptakis-(6-azido-6-deoxy)-β-cyclodextrin (β-CDN_3_, **2**) by the click chemistry reaction. Finally, reduction of the residual nitrogen groups of CNT-CDN_3_ (**3**) and treatment with excess HCl provided the protonated forms of the target CNT-CD (**5**) (Figure 2). The amount of free amino groups, measured spectroscopically using Kaiser’s colorimetric test (Figure S1),^22^ was found to be 0.607 mmol g^–1^, approximately consistent with six amine groups per CD unit. Thus, the amount of grafted CDs was found to be 0.101 mmol g^–1^. The functionalized CNTs were characterized by several techniques, including TGA, micro-Raman, SEM, and XPS analyses (Figures 3, 4 and S2). The unequivocal evidence of the grafted organic groups on the modified CNTs was provided by TGA (Figure 3A). Indeed, by analyzing the thermal degradation trend of CNT-CD and its progenitor compounds, the chemical modification of the CNT structure induced by organic functionalization is evident. In particular, the thermal degradation of pristine CNTs shows enhanced thermal stability with nonsignificant mass loss up to 500 °C, which is typical behavior of unmodified CNT-based materials, whereas derivatized CNT-Alk (**1**) shows an initial degradation starting at about 176 °C, probably attributable to decarboxylation phenomena, followed by a continuous degradation phase that leaves a residue (68.8%) at 800 °C. After functionalization of CNT-Alk with the CD azide derivative, CNT-CDN_3_ (**3**) shows a different degradation course due to the presence of the CD derivative and the reactive azide moiety grafted onto the CD units. The degradation evolves with an initial step at about 150 °C and subsequent unresolved multidegradation steps at 800 °C that leaves a residue of 24.4%. Finally, CNT-CD (**5**) shows a more stable thermal course, as expected considering the transformation of azide groups to protonated amine species, with two degradation phases beginning at about 138 and 364 °C, respectively, leaving a residue of 63.3% at 800 °C.

Information about the microstructure of CNT-CD was deduced by analyzing the highest wavelength region of the micro-Raman spectra (Figure 3B), which is dominated by the bands associated with the C sp^2^ vibration modes. The G-band (at 1582 cm^–1^ in graphite), originating from the in-plane stretching mode of all pairs of hybridized C sp^2^ atoms, is the fingerprint of the graphitic crystal arrangement. The D band (at 1346 cm^–1^ for excitation at 532 nm), related to the breathing mode of the hexagonal C rings, is activated by the presence of disorder in the aromatic structure.^24,25^ One part of the G band (about 1590 cm^–1^) is associated with the vibrations of carbon atoms along the axis of the tube (LO phonon mode), whereas the other (about 1570 cm^–1^) is associated with the vibrations of carbon atoms along the circumferential direction of CNTs (TO phonon mode).^26,27^ In addition, a second-order mode between 2450 and 2680 cm^–1^ is assigned to the first overtone of the D mode (generally called the G′ band). The expected increase in the *I*_D_/*I*_G_ ratio, due to increased structural disorder in the graphitic lattice after functionalization,^28^ has not been observed. However, according to the literature,^29^ this trend can be attributed to the suppression of the signal from the outer walls of the CNTs, which are chemically modified by the grafted CDs, with the unmodified inner tube walls contributing only to the Raman intensity. Simultaneously, in the functionalized sample, the slight increase toward the lower wavenumbers of the D bandwidth is indicative of a disorder process due to the presence of CD/nanotube interactions.

The functionalization of CNTs with CDs was also confirmed by XPS analysis (Figure 4). As expected, CNT-Alk, CNT-CDN_3_, and CNT-CD showed different contents of C, O, and N depending on their functionalization (Table S1). The carbon contents of CNT-CDN_3_ and CNT-CD samples (about 60–66%) are comparable but lower than those of CNT-Alk (83%). The C 1s profiles are composed of contributions at 284.5 eV due to C–C bonds and contributions at binding energies above 285 eV attributed to oxygen- or nitrogen-bonded carbon in various configurations (see Figure 4a–c), particularly C–OH (285.4 eV), C–O–C or C–N (286.1 eV), C=O (286.9 eV), and O–C=O (287.8 eV)^30^ (see Figure 4a–c). Nitrogen (from 3.2 to 9.2%) and oxygen (from 13 to 25%) increase for both CNT-CDN_3_ and CNT-CD compared with CNT-Alk, confirming the derivatization of CNT with β-CD. In detail, the N 1s spectra are characterized by a broad band with three contributions located at 399.0, 400.1, and 401.5 eV and assigned to N–C (sp^2^), N–C (sp^3^), and protonated amine (−NH_3_^+^), respectively. Further, in the 404–407 eV region, two additional bands due to NO_*x*_ (NO^–^_2_ and NO^–^_3_) species^31^ are visible (Figure 4d–f), which is in good agreement with the highest amount of carbon–oxygen species evidenced by the C 1s profile (Figure 4a–c) and the change of azide groups in the protonated amine species, previously discussed. Table S2 shows the results derived from the deconvolved C 1s and N 1s profiles, respectively.

SEM analysis showed pristine CNTs and CNT-Alk spatially dispersed in random orientations with a diameter of less than 20 nm (Figure S2a,b). Functionalization with CDs, by click reaction, produced massive aggregates with marked spatial inhomogeneity (Figures S2c,d and S3). SEM images of CNT-CDN_3_ and CNT-CD evidenced diffuse and hazy contrast due to the amorphous CD moieties (Figures S2 and S3).

### 3.2 Electrochemical Investigation

**Figure 5:**
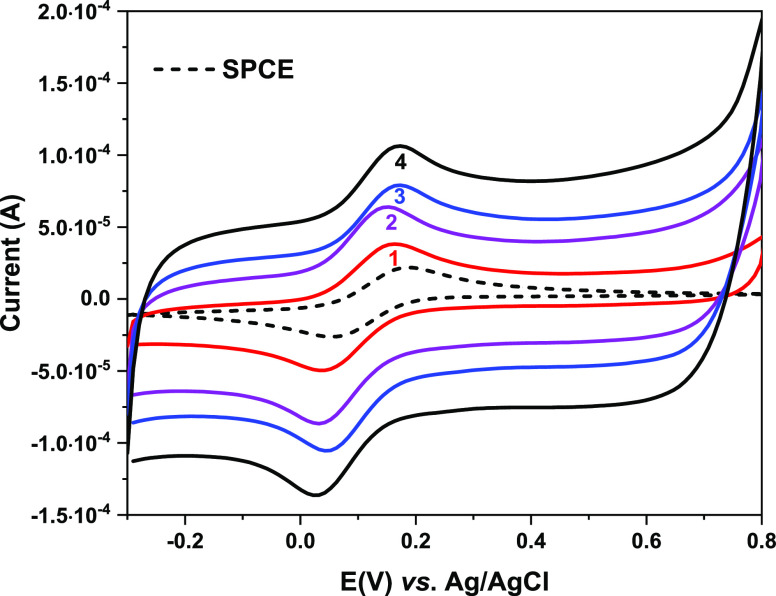
CVs of [Fe(CN)_6_]^3–^ (1 mM) in KCl (0.1 M) obtained on bare SPCE (dashed line) and SPCE/CNT-CD, after successive castings of a dispersion of CNT-CD in DMF (solid lines, 1–4). Scan rate: 0.1 V s^–1^.

As outlined in Section 2.3, 1 μL of CNT-CD dispersion was cast four times on the electrode surface, and after each casting, the electrochemical response on SPCE/CNT-CD was analyzed at different levels using [Fe(CN)_6_]^3–^ as the redox probe. The cyclic voltammograms (CVs) are depicted in Figure 5, together with that obtained on bare SPCE (dashed line). By increasing the layering of CNT-CD on the SPCE, signal amplification is observed due to the deposition of CNT conductive material that induces capacitive behavior on the electrode surface. CNT-CD dispersion was cast up to 4 μL, after which no further change in CV was observed, indicating complete coverage of the surface. The slight increase in Δ*E*_p_ from the bare SPCE (115 mV) to the modified one (130 mV) could be due to the repulsive interactions between the anionic [Fe(CN)_6_]^3–^ and carboxylate groups on the surface of the SPCE/CNT-CD, resulting in electron transfer hindrance.

**Figure 6:**
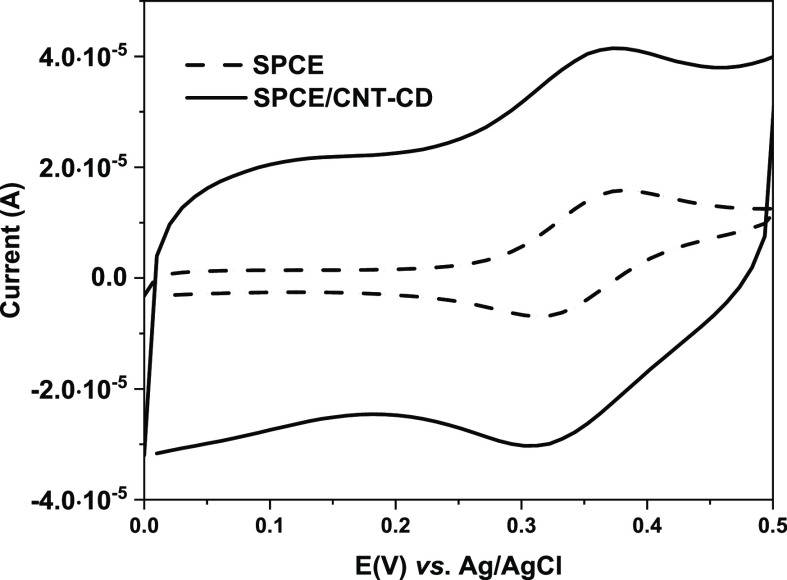
CVs of FcCAR (1 mM) in KCl (0.1 M), obtained on bare SPCE (dashed line) and SPCE/CNT-CD (solid line). Scan rate: 0.1 V s^–1^.

**Figure 7:**
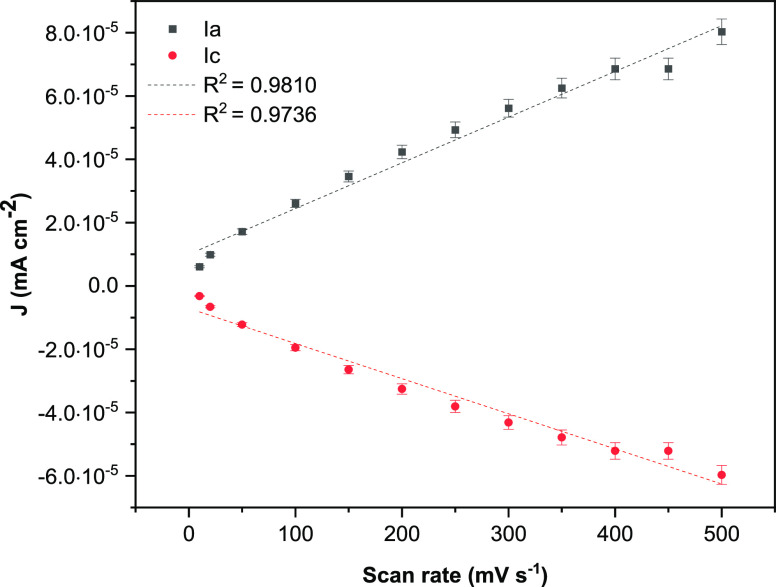
Dependence of anodic (*I*_a_) and cathodic (*I*_c_) peak currents on the scan rate for self-assembled FcCAR on SPCE/CNT-CD in KCl (0.1 M).

Therefore, the electrochemical behavior of FcCAR on SPCE/CNT-CD compared with that of bare SPCE was analyzed. In both cases, FcCAR undergoes a one-electron-reversible redox process. Figure 6 shows the CVs of the electrochemical response of FcCAR (1 mM) in KCl (0.1 M) on bare SPCE and SPCE/CNT-CD. The CVs exhibit a pseudorectangular shape, characteristic of the capacitive behavior of the electric layer of CNT-CD. The capacitive current increases with the oxidation degree of CNT-CDs, due to the presence of functional groups on their surface. Moreover, in the CVs of bare SPCE and SPCE/CNT-CD, anodic and cathodic peaks are observed at 0.373 V and 0.312 V (vs. Ag/AgCl), respectively. These values do not shift due to the modification strategy, and the low Δ*E*_p_ (61 mV) indicates a rapid electron transfer process of Fc on the CNT-CD-modified surface. The peak currents vary linearly with the scan rate, indicating that the redox process is probably due to confined redox species on the CNT-CD surface (Figure 7). The anodic and cathodic peak currents obtained on SPCE/CNT-CD are higher than those on bare SPCE, pointing out better electrocatalytic activity toward this molecule. The best-resolved response for FcCAR was obtained on SPCE/CNT-CD compared to that on SPCE.

**Figure 8:**
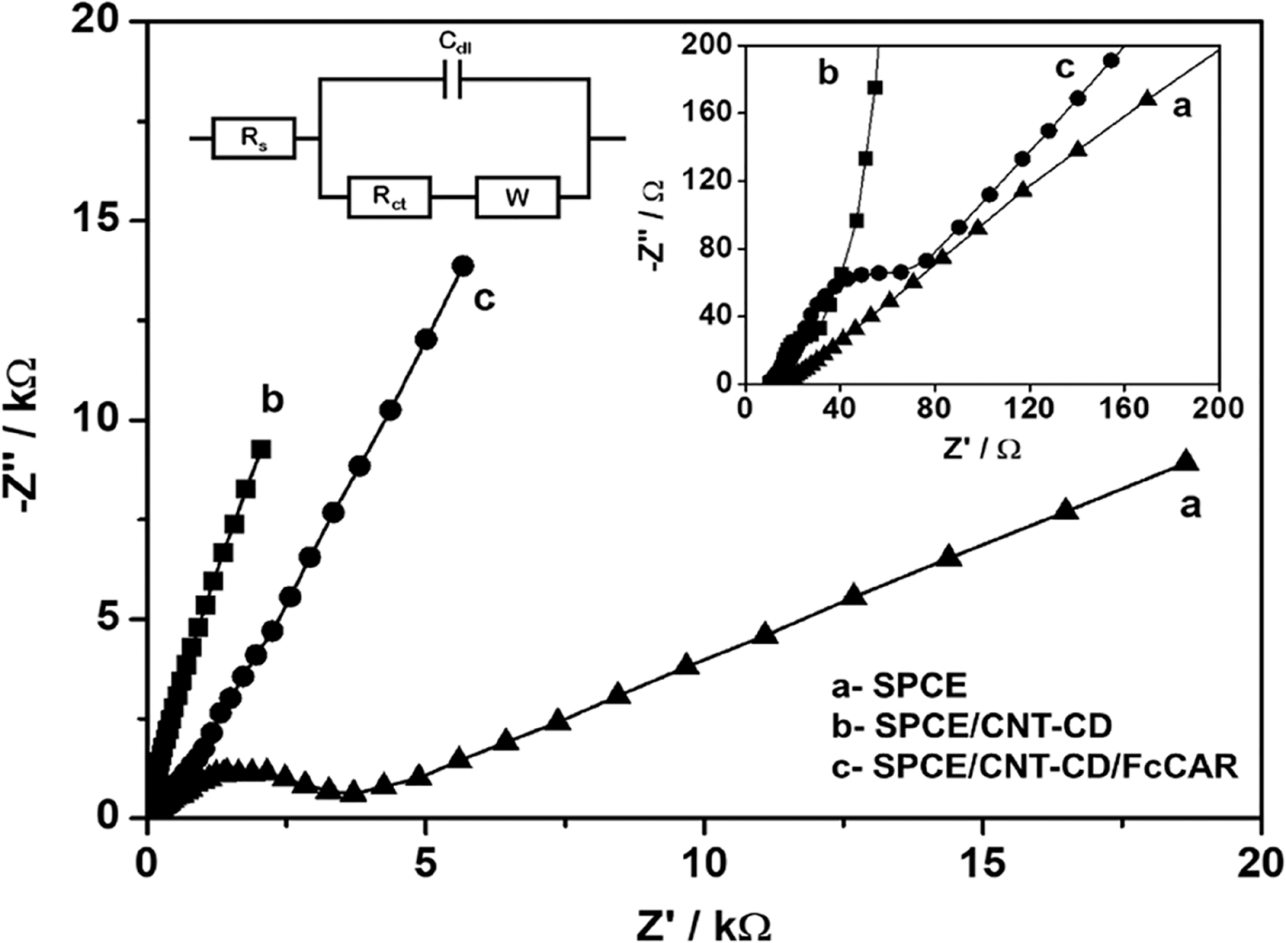
Nyquist plots (in 1 mM [Fe(CN)_6_]^3–/4–^ in 0.1 M KCl) corresponding to the sequential deposition of CNT-CD and FcCAR on the SPCE. Insets show the high-frequency region of the plot and the equivalent circuit used to calculate the impedance parameters.

To characterize the sequential deposition of CNT-CD and FcCAR on the bare SPCE, EIS measurements in [Fe(CN)_6_]^3–/4–^ were used. The Nyquist plot for the bare and modified electrodes, together with the equivalent circuit model used to fit the impedance spectra, is shown in Figure 8. The impedance response of the CNT-CD-modified electrodes shows a high slope, typical of capacitive behavior resulting from the deposition of carbon nanotubes, with *C*_dl_ values of 950 and 535 mF for SPCE/CNT-CD and SPCE/CNT-CD/FcCAR, respectively (*C*_dl_ of bare SPCE was 1.2 mF). The charge transfer resistance (*R*_ct_), corresponding to the semicircular part of the high-frequency spectra, initially decreased from 2.9 kW for the bare electrode to 44 W for SPCE/CNT-CD, indicating a much faster electron transfer of the [Fe(CN)_6_]^3–/4–^ probe into the more conductive electrode surface, followed by an increase to 105 W after interaction with FcCAR, which slightly blocks this process and increases the charge transfer resistance from the electroactive probe.

**Figure 9:**
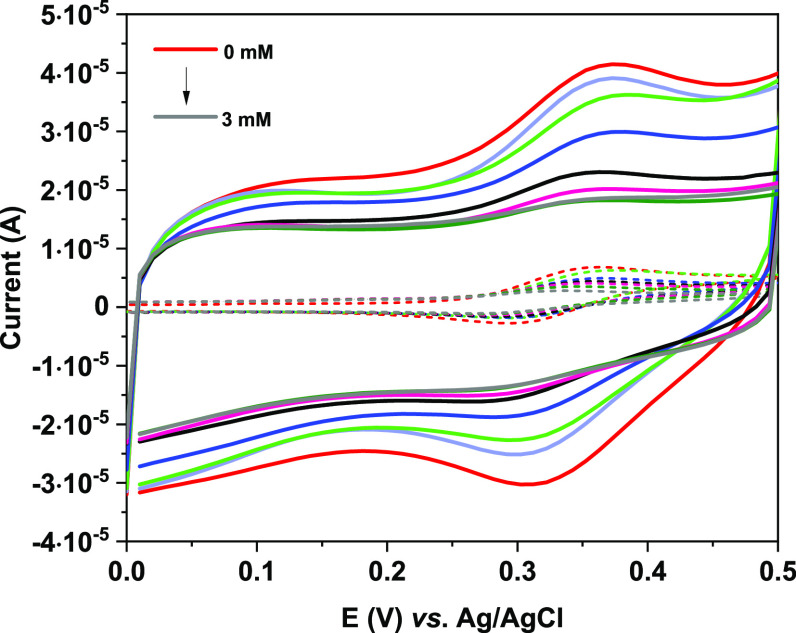
CVs obtained by titrating a solution of FcCAR (1 mM) in KCl (0.1 M) with Hg(II) (pH = 7) on SPCE/CNT-CD (solid line) and SPCE (dashed line).

Once we had analyzed the electrochemical behavior of the SPCE/CNT-CD/FcCAR system, we evaluated its use as a sensor of Hg(II), considering the strong affinity of FcCAR toward this metal ion.^19^ First, electrochemical measurements were performed by titrating solutions containing FcCAR with those of Hg(II), previously prepared in MOPS buffer (pH 7), using KCl (0.1 M) as the supporting electrolyte. For instance, Figure 9 shows a comparison between the CVs obtained on SPCE/CNT-CD and those on SPCE.^19^ In both cases, no deposition phenomena were observed, and the FcCAR signals decreased as the concentration of Hg(II) increased. This effect could be due to the coordination of Hg(II) to the dipeptide moiety of FcCAR. Modifying the SPCE with CNT-CD/FcCAR resulted in a 20-fold increase in the signal toward Hg(II), and the modified electrode showed a very rapid response, as confirmed by amperometric measurements (Figure S4).

**Figure 10:**
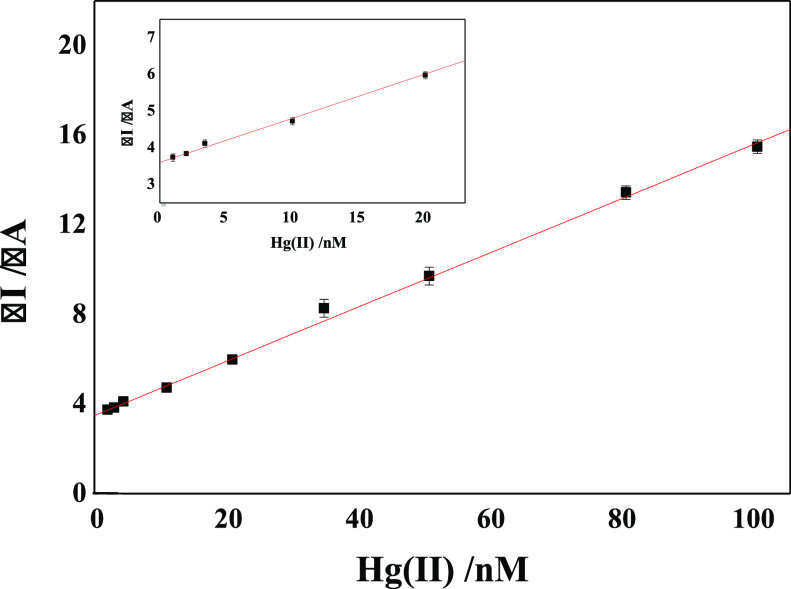
Linear range of 1–100 nM concentration of the anode peak current (in the inset, the magnified region of 1–20 nM).

The analytical parameters of SPCE/CNT-CD/FcCAR as a sensor of Hg(II) were evaluated by DPV using standard solutions of Hg(II) in the concentration range of 0.5 nM–10 mM. The calibration curve, constructed from the change in the anode peak current before and after the addition of Hg(II), displayed a linear dependence on concentration in the range of 1–100 nM, as shown in Figure 10 (*R*^2^ = 0.994) with a sensitivity (assumed as the slope of the calibration curve) of 0.12 μA/nM. The LOD was calculated as 0.50 nM based on the 3σ/slope approach.^32^ The initial value of the linear range (1 nM) was established by the first analyte concentration that can be accurately determined (LOQ).

To evaluate the repeatability and reproducibility of SPCE/CNT-CD/FcCAR as a Hg(II) sensor, DPV measurements were performed on a solution containing 20 nM Hg(II) (in 0.1 M KCl). Repeatability was established based on the relative standard deviation (RSD %) of 5 consecutive DPV measurements made with the same device. Unfortunately, the repeatibility is not high (RSD 32%), probably due to the deposition of some electroactive byproducts of Hg formed during the redox process. Reproducibility was determined by using different electrodes. The current responses floated from 8.149 to 8.756 μA with a relatively small RSD of 2.8%, showing that the sensor has appreciable reproducibility. A preliminary stability study showed a decrease in FcCAR signal of only 2% after storing the SPCE/CNT-CD/FcCAR electrode at room temperature in KCl (0.1 M) for 2 weeks (Figure S5).

## 4 Conclusions

To sum up, in this work, we exploited the peculiar features of cationic CD multiwalled CNTs (MW-CNT-CDs) to design nanoassembly of CNT-CD and FcCAR with toxic heavy ion sensing capability. The nanoassembly conveys both the outstanding affinity and chelating ability toward Hg(II) ions of the FcCAR ligand and the ability of carbon nanomaterials to increase electrical conductivity and electrode surface area by changing their shape and structure. CNT-CDs were synthesized by a two-step procedure involving the click reaction between reactive alkyne and azide fragments present on the sidewalls of MW-CNTs and CDs respectively, and the subsequent reduction of the residual azide groups. The morphology, chemical composition, and structure of CNT-CD were studied by micro-Raman, XPS, SEM, and TGA.

The electrochemical response of SPCE/CNT-CD was analyzed by CV using [Fe(CN)_6_]^3–^ (1 mM) in KCl (0.1 M), and compared with that previously studied on bare SPCE.^19^ The affinity of FcCAR toward mercury, a toxic metal included in the list of “priority heavy metals” for its adverse effects on human health and the environment, was also analyzed on both electrodes. The signals of FcCAR decreased as the concentration of Hg(II) increased, whereas the CNT-CD/FcCAR nanoassembly was found to improve the current response and sensitivity of SPCE toward the metal. In particular, for the same concentration of Hg(II) ions, a higher linear range and an increase in peak current, especially in the anodic peak signal (up to 84%), was observed for SPCE/CNT-CD compared with bare SPCE. In light of these results, we evaluated the analytical parameters of SPCE/CNT-CD/FcCAR as a sensor of Hg(II), obtaining a linear concentration range of 1–100 nM, with sensitivity of 0.12 μA/nM and LOD of 0.50 nM. These results, compared with more recent work using modified SPCE as a Hg(II) sensor (Table S3), highlight the importance of innovative nanohybrid systems, which not only combine the properties/characteristics of the starting components but also give rise to new properties due to the synergistic action of the native ones and, therefore, are able to increase the electrochemical response and facilitate the engagement of the recognition element on the electrode surface.

## Data Availability

Data will be made available on request.

## Supporting Information Available

Reagents; Kaiser test protocolpage; synthesis of carbon nanotubes modified with alkyne terminated group (CNT-Alk); Figure S1: UV–vis absorption spectra of the CNT-CD-NH2 and CNT-CD-N3 samples; Figure S2: SEM images of CNTs, CNT-Alk, CNT-CDN3 and CNT-CD; Figure S3: SEM images of CNT-CD; Figure S4: amperometric response of the electrodes before and after addition of Hg(II); Figure S5: preliminary stability study; Table S1: atomic species expressed in percentage for all the investigated samples; Table S2: XPS spectral analyses of the investigated samples; Table S3: comparison with the most recent Hg(II) sensors using modified SPCE electrodes (PDF)
