# Supplementary material for: Screen-Printed Carbon Electrodes with Cationic Cyclodextrin Carbon Nanotubes and Ferrocenyl-Carnosine for Electrochemical Sensing of Hg(II)
Source: ACS Appl Nano Mater. 2023 Sep 11;6(18):17187–95. doi: 10.1021/acsanm.3c03480 (PMC10520977; doi:10.1021/acsanm.3c03480)
Supplement: Supplementary file 1 — an3c03480_si_001.pdf [file an3c03480_si_001.pdf]

SUPPORTING INFORMATION

Screen-Printed Carbon Electrodes (SPCEs) with Cationic Cyclodextrin Carbon Nanotubes and Ferrocenyl-Carnosine for Electrochemical Sensing of Hg(II)

Chiara Abate†[1], Giulia Neri†[1], Angela Scala[1], Placido Giuseppe Mineo[2], Enza Fazio[3], Antonino Mazzaglia[4], Alex Fragoso[5], Ottavia Giuffrè,[1] Claudia Foti, [1*] Anna Piperno[1]

[1]*Department of Chemical, Biological, Pharmaceutical and Environmental Sciences, University of Messina, Viale F. Stagno d'Alcontres 31, 98166 Messina, Italy (cfoti@unime.it]*
[2]*Department of Chemical Sciences, University of Catania, V.le A. Doria 6, 95125 Catania, Italy (gmineo@unict.it)*
[3]*Department of Mathematical and Computational Sciences, Physical Sciences and Earth Sciences, University of Messina, Viale F. Stagno d'Alcontres 31, 98166 Messina, Italy; enfazio@unime.it (E.F.)*
[4]*National Council of Research, Institute for the Study of Nanostructured Materials (CNR-ISMN), URT of Messina c/o Department of Chemical, Biological, Pharmaceutical and Environmental Sciences, University of Messina, V.le F. Stagno d'Alcontres 31, 98166 Messina, Italy 98122 Messina, Italy*
[5]*Inferfibio Research Group, Departament d'Enginyeria Química, Universitat Rovira i Virgili, Avinguda Països Catalans 26, 43007 Tarragona, Spain*

Contents

Reagents	page S2
Kaiser test protocolpage	page S2
Synthesis of carbon nanotubes modified with alkyne terminated group (CNT-Alk)	page S2
UV-Vis absorption spectra of the CNT-CD-NH_2 and CNT-CD-N_3 samples	page S3
SEM images of: (a) CNTs, (b) CNT-Alk, (c) CNT-CDN_3 and (d) CNT-CD	page S3
SEM images CNT-CD page	page S4
Amperometric response of the electrodes before and after addition of Hg(II)	page S5
Preliminary stability study	page S5
Atomic species expressed in percentage for all the investigated samples	page S6
XPS spectral analyses of the investigated samples	page S6
Comparison with the most recent Hg(II) sensors using modified SPCE electrodes	page S7

Reagents

Potassium chloride (KCl, extra pure, Scharlau) and sulfuric acid (95-98% EPR, Panreac) were used without purification. Potassium ferricyanide(III) ($K_3Fe(CN)_6$, 99+%), ferrocenecarboxylic acid (>96.0%) and anhydrous DMF were purchased from Sigma Aldrich (Spain) and used as received. Ferrocenyl-carnosine was synthetized as reported in (C. Abate et al, *Dalton Trans.* **2023**, *52* (12), 3699-3708). Mercury(II) chloride solutions were prepared by weighing and dissolving the respective product (99.5%, Riedel-de Haën) in MOPS buffer containing 40 mM 3-(N-morpholino)propanesulfonic acid, 10 mM sodium acetate and 1 mM EDTA and adjusted to pH 7. All solutions were prepared with double distilled water (18.2 MΩ cm^{-1}) obtained from a Milli-Q® system (Millipore, Madrid, Spain).

Synthesis of carbon nanotubes modified with alkyne terminated group (CNT-Alk)

Carbon nanotubes modified with alkyne terminated group (CNT-Alk) were synthetized as reported in Ref.22, following a brief description of the synthetic strategy.

To increase the hydrophilicity of commercial CNTs, they were oxidized by a treatment with sulfuric acid: nitric acid solution. To reach a homogenous dispersion, commercial CNTs (500 mg) were sonicated (60W, 35 kHz) in 100 mL of sulfuric acid:nitric acid (3:1 v/v, 98% and 69%respectively), then the mixture was stirred at 60° C for 6 h. Afterwards, 500 mL of MqW were added and into the reaction mixture, which was filtered under vacuum (0.1 µm Millipore membrane) and washed with MqW until neutral pH. The residue was dried at 60 °C to recover 490 mg of CNTs-Ox.

400 mg of CNTs-Ox were dispersed in *o*-dichlorobenzene (100 mL) by sonication treatment at room temperature. 2 g of *p*-(2-propylnyloxy)-benzamine were solubilized in 200 mL of anhydrous acetonitrile and added into reaction mixture, which was sonicated for 30 min at room temperature. Then 3.0 mL of isoamylnitrite were added and the reaction was stirred at 60◦C for 24 h under inert atmosphere. The mixture was cooled, diluted with ethanol and filtered under vacuum (0.1 µm Millipore membrane). The residue underwent to washing cycles by using several solvents (water, ethanol, chloroform) and it was dried at 60◦C to give 390 mg of CNT-Alk.

Kaiser test protocol

The commercial Kaiser's test kit consists of three solutions: (a) 0.5 g mL^{-1} phenol in absolute EtOH; (b) 2 mL of 1 mM potassium cyanide (aqueous solution) dissolved in 98 mL of pyridine; (c) 0.05 g mL^{-1} ninhydrin in absolute EtOH. Briefly, 0.5 mg of CNT-CD was treated sequentially with 75 µL of solution (a), 100 µL of solution (b) and 75 µL of solution (c). The dispersion was sonicated in a water bath and then heated at 120 C for 5 min, diluted with 4750 µL of absolute EtOH and centrifuged at 14.000 rpm. The absorbance at 570 nm of the supernatant was correlated with the amount of free amine groups (mmol g^{-1}) on the CNT-CD using the following equation:

[free amines (mmol/g)] = ([Abs] x dilution x 1000)/(ε x sample weight x optical path)

where the dilution was set at 5 mL, the optical path was 1 cm; the sample weight was 0.5 mg, and the extinction coefficient was 15.000 mol^{-1} L cm^{-1}.

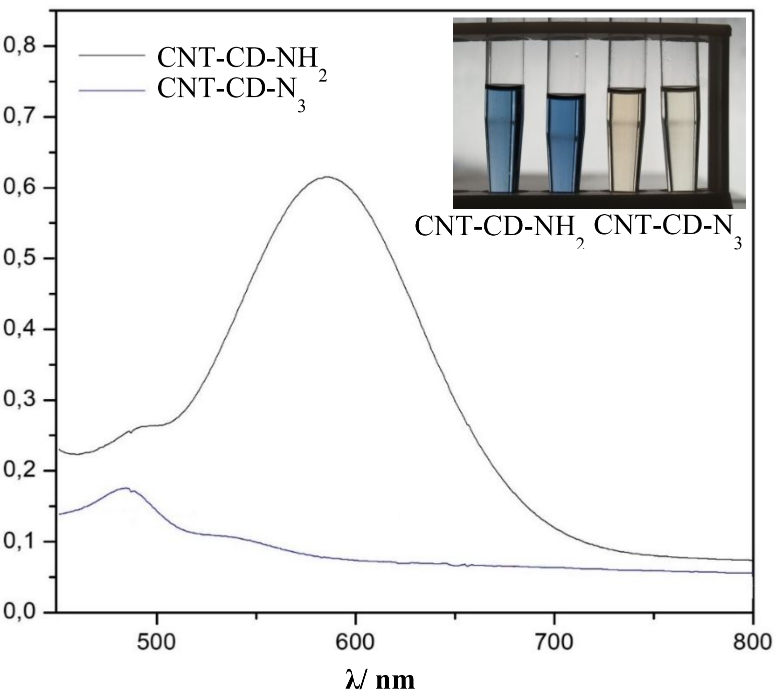

Fig. S1. UV-Vis absorption spectra of the CNT-CD-NH_2 and CNT-CD-N_3 samples

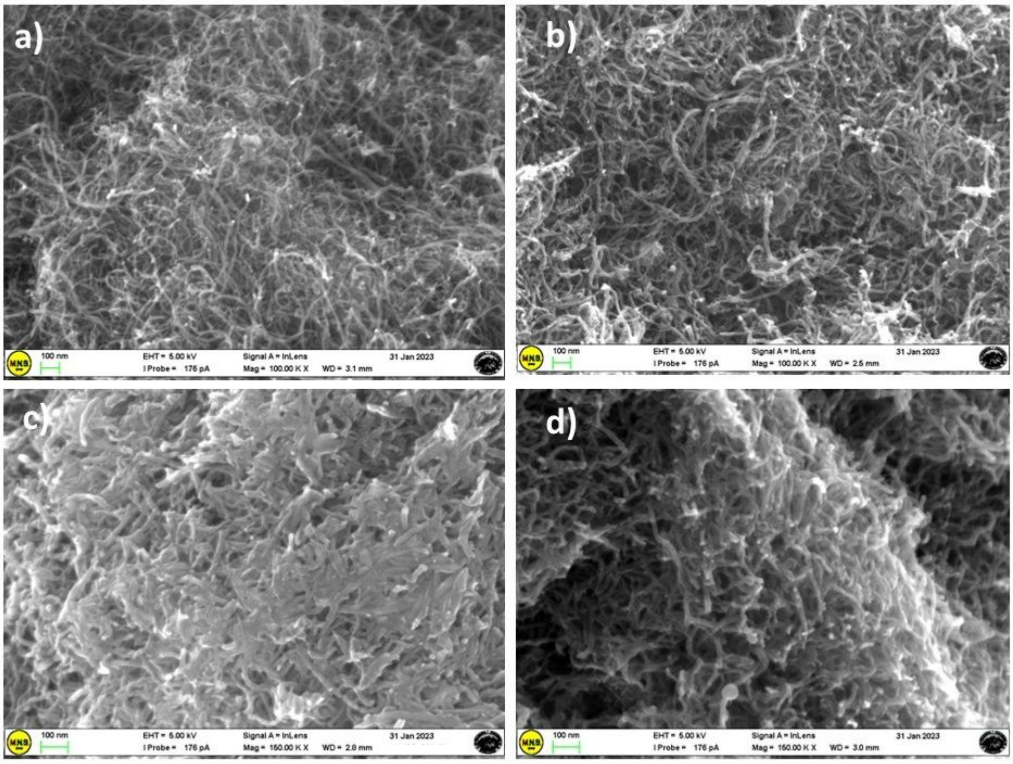

Fig. S2. SEM images of: (a) CNTs, (b) CNT-Alk, (c) CNT-CDN_3 and (d) CNT-CD.

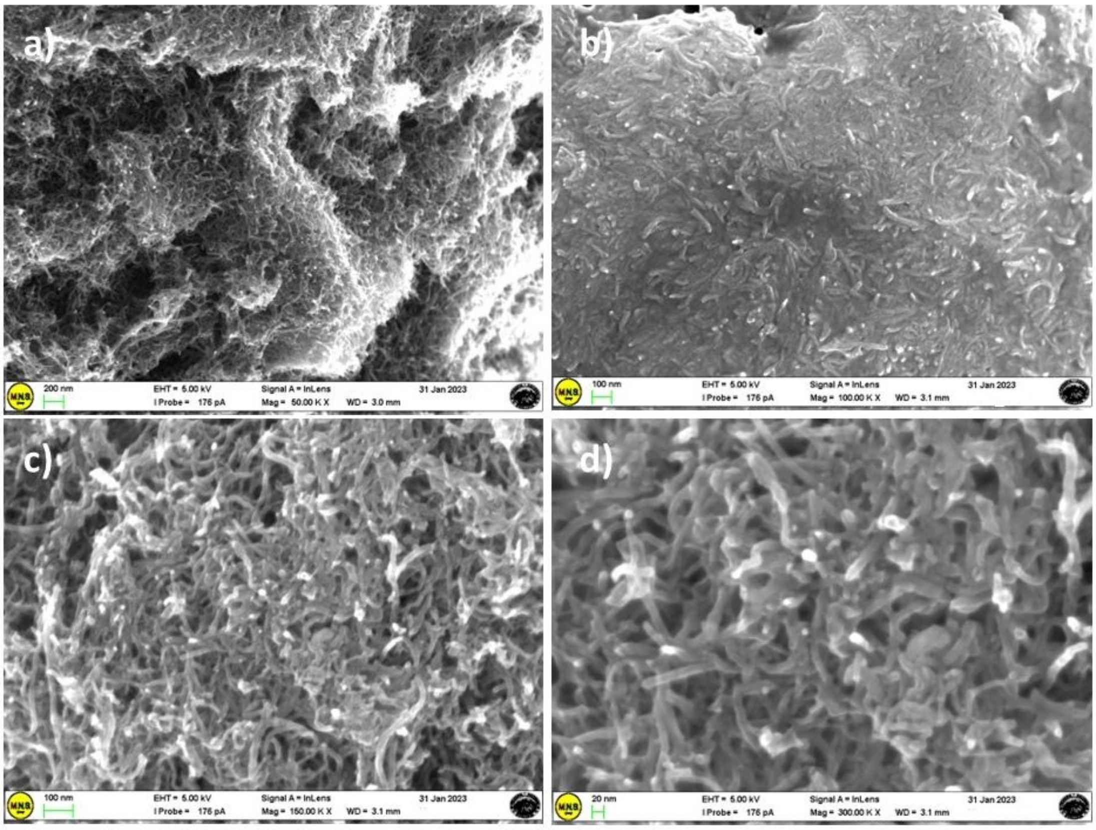

Fig. S3. SEM images of CNT-CD.

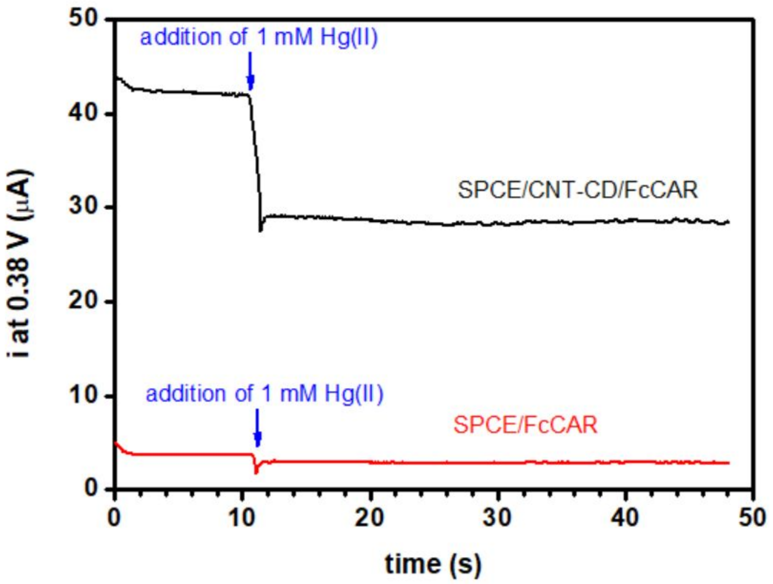

Fig. S4. Amperometric response at +0.38V (*vs*. Ag/AgCl) of the indicated electrodes before and after addition of Hg(II).

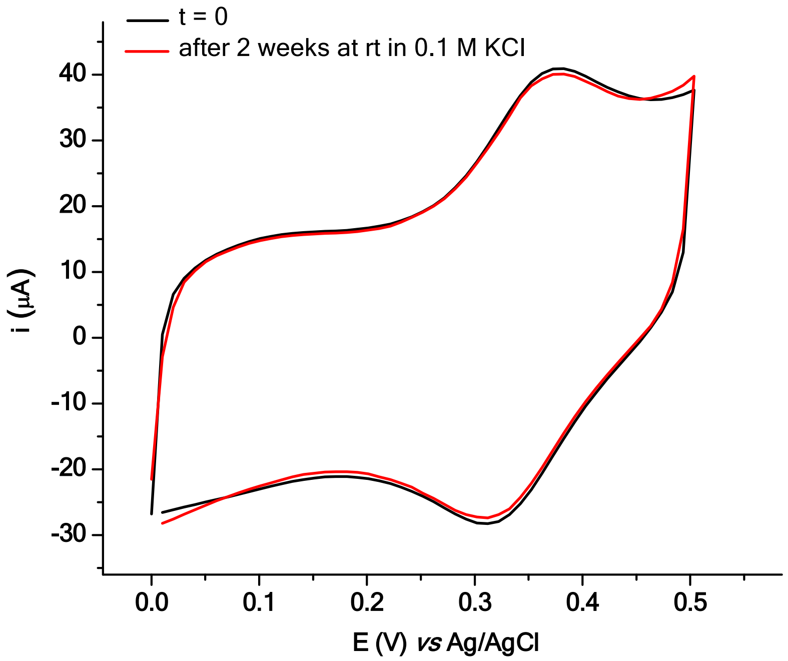

Fig. S5. CVs of SPE/CNT-CD/FcCAR electrode before and after 2 weeks of storage at room temperature in KCl (0.1 mol L^{-1}).

Table S1. Atomic species expressed in percentage for all the investigated samples.

	C (%)	O (%)	N (%)
CNT-Alk	83.4	13.4	3.2
CNT-CDN$_3$	61.5	25.3	9.2
CNT-CD	72.7	21.8	5.5

Table S2. XPS spectral analyses of the investigated samples, according to the assignements reported in the text.

C 1s lineshape	C-C (%)	C-OH (%)	C-O-C or C-N (%)	C=O (%)	O–C=O (%)	π-π (%)
CNT-Alk	62	15.1	10.4	5.0	4.0	3.5
CNT-CDN$_3$	28	15.9	31.5	14.7	6.9	3.0
CNT-CD	41.3	17.0	25.7	7.6	4.1	4.3

N1s lineshape	N-C sp^2 (%)	N-C sp^3 (%)	NH_3^+ (%)	NO^-_2 (%)	NO^-_3 (%)
CNT-Alk	27.1	42.7	25.4	0.2	4.6
CNT-CDN$_3$	12.6	48.3	20.7	17.9	0.5
CNT-CD	2.8	64.5	28.0	0.9	3.8

Table S3. Comparison with the most recent Hg(II) sensors using modified SPCE electrodes

SPCE modified	Electrochemical sensing of Hg(II)	Linear Hg concentration range	Detection limit	Ref
(S, O-GCN)[a]	differential pulse anodic stripping voltammetry (DPASV) in 0.1M KCl	0.05-390 nM	13 pM	1
BiM/GR[b]	differential pulse voltammetry (DPV) in 0.05 mol L^{-1} PBS (pH 7)	20–149000 nM	5 nM	2
AgNS[c]	Cyclic voltammetry (CV) and differential pulse stripping voltammetry (DPSV) in 0.1M acetate buffer (pH 4.4)	5-100ppb	0.7 ppb	3
AgNWs/HPMC/CS/Urease[d]	CV in 0.1M KCl (pH 3-11)	5-25 μM	3.94 μM	4
Au-PANOA[e]	Square wave anodic stripping voltammetry (SWASV) in tris buffer (pH 6)	0.8-12 nM	0.23	5
ZnO/Ag/ZIF-8/[f]	DPV in 0.05 mol L^{-1} PBS (pH 7)	500–140000	40 nM	6
Bi NPs@GO-MWCNTs/[g]	DPV in 0.01 mol L^{-1} PBS (pH 7.2)	0.001-5 mmol L^{-1}	2.57 10^2 μA L μmol^{-1} cm^{-2}	7

[a] Sulfur and oxygen-incorporated graphitic carbon nitride (S, O-GCN) linked poly(1,3,4-thiadiazole-2,5-dithiol) film (PTD) through thioester linkage; [b] Bismuth Molybdate/Graphene; [c] butterfly-shaped silver nanostructure; [d] Silver Nano wires/HPMC/Chitosan/Urease; [e] gold-decorated polymer nanofibers; [f] Zinc oxide/silver/metal organic frame work; [g]composite based on a polyaniline (PANi) framework doped with bismuth nanoparticle@graphene oxide multi-walled carbon nanotubes (Bi NPs@GO-MWCNTs)

Refs. (1) Manikandan, R.; Pugal Mani, S.; Selvan, K. S.; Yoon, J.-H.; Chang, S.-C. *Food Chemistry* **2023**, *425*, 136483. (2) Sakthi Priya, T.; Chen, T.-W.; Chen, S.-M.; Kokulnathan, T.. *ACS Applied Nano Materials* **2022**, *5* (9), 12518-12526. (3) Naseri, M.; Mohammadniaei, M.; Ghosh, K.; Sarkar, S.; Sankar, R.; Mukherjee, S.; Pal, S.; Ansari Dezfouli, E.; Halder, A.; Qiao, J.; et al.. *Electroanalysis* **2023**, *35* (2), e202200114. (4) Saenchoopa, A.; Klangphukhiew, S.; Somsub, R.; Talodthaisong, C.; Patramanon, R.; Daduang, J.; Daduang, S.; Kulchat, S. A *Biosensors* **2021**, *11* (10), 351. (5) Narouei, F. H.; Livernois, L.; Andreescu, D.; Andreescu, S. *Sensors and Actuators B: Chemical* **2021**, *329*, 129267. (6) Arabbani, F. K.; Vasu, D.; Sakthinathan, S.; Chiu, T.-W.; Liu, M.-C. *Electroanalysis* **2023**, *35* (3), e202200284. (7) Bao, Q.; Li, G.; Yang, Z.; Pan, P.; Liu, J.; Chang, J.; Wei, J.; Lin, L. *Chinese Chemical Letters* **2020**, *31* (10), 2752-2756.
